# Effects of Cement and Emulsified Asphalt on Properties of Mastics and 100% Cold Recycled Asphalt Mixtures

**DOI:** 10.3390/ma12050754

**Published:** 2019-03-05

**Authors:** Yanan Li, Yuchao Lyv, Liang Fan, Yuzhen Zhang

**Affiliations:** 1 College of Chemical Engineering, China University of Petroleum, Qingdao 266580, Shandong Province, China; liyanan@s.upc.edu.cn (Y.L.); B15030098@s.upc.edu.cn (Y.L.); 2 Road Engineering Laboratory, Shandong Transportation Research Institute, Jinan 250000, Shandong Province, China; fanliang@sdjtky.cn

**Keywords:** cold recycled asphalt mixture, reclaimed asphalt pavement, mastic, rheological properties, emulsified asphalt, cement

## Abstract

Cold recycled asphalt mixtures (CRAM) are a cost-effective and environmentally-friendly way to reuse reclaimed asphalt pavement (RAP). This paper evaluates the rheological properties and microstructure of mineral filler-asphalt mastic, mineral filler-residue mastic, and cement-residue mastic. Then, based on the premise of using 100% RAP with a gradation that was determined experimentally, the effects of emulsified asphalt and cement on the porosity, indirect tensile strength, tensile strength ratio, dynamic stability, and mechanical properties of CRAM were evaluated. It was found that the rheological properties and cohesive coefficient of the cement-residue mastic varied differently to those of the first two types of mastic and the results show that the addition of cement can greatly improve the interfacial bonding between binders and fillers in the mastic, thereby improving the water damage resistance and high-temperature stability of CRAM. The relationships between cement content and the dynamic modulus and phase angle of CRAM are different to that for emulsified asphalt obviously. In addition, under certain conditions, the properties of CRAM can meet the requirements of relevant technical specifications for its application to subsurface layer of pavement. Hence, the use of 100% RAP in CRAM may be feasible.

## 1. Introduction

Asphalt pavement is subject to aging, and many pavements require servicing after 10–20 years. In order to ensure the performance and safety of roads, many pavements need to be rebuilt, resulting in the production of tens of thousands of tons of reclaimed asphalt pavement (RAP) each year. Therefore, discovering ways of making better use of RAP is important [1,2].

Reclaimed materials have been used in pavement construction for years. Nowadays, RAP’s use is increasing rapidly with the advancement of material processing equipment and mix design technologies [3]. Much research has focused on the use of RAP in hot recycled asphalt mixture (HRAM) [4,5,6]. It has been proven that HRAM with RAP contents of 10% to 30% perform similarly to virgin hot-mix asphalt mixture (HMA) [7,8]. However, due to its higher mixing temperature, it has a shorter period in which it can be used for construction during the year. Additionally, it has high fuel consumption and produces many toxic fumes during the paving process, which are not conducive to energy conservation, emission reduction, and environmental protection. Unlike HRAM, cold recycled asphalt mixture (CRAM) can further solve the above problems to a large extent [9]. With the increasing concerns of energy conservation and environmental protection, CRAM has been increasingly applied for pavement rehabilitation.

CRAM is regarded as a semi-flexible material, which is different from cement concrete and HMA. CRAM has lower fatigue life as compared with ordinary HMA, especially at high strain mode and high stress model [10]. Cement, lime, and fly ash are increasingly used as additives to improve the properties of CRAM [11,12,13,14]. More and more research has focused on the design and performance evaluation of CRAM [15,16,17,18]. Despite this, there are few studies on the properties of CRAM when their aggregate gradation uses 100% RAP and no new aggregate is added. Therefore, this paper investigated the influences of emulsified asphalt and cement contents on the properties of CRAM, such as porosity, indirect tensile strength (ITS), freeze–thaw splitting strength ratio, and dynamic modulus, with gradations using 100% RAP.

Asphalt pavement material can be seen as a multi-level distributed system with a spatial network structure. This multi-level dispersion system consists of three levels: a micro-dispersion system (mastic) in which the fillers are dispersed in the asphalt binder, a fine-dispersion system (mortar) in which the fine aggregates are dispersed in the mastic, and a coarse-dispersion system in which the coarse aggregates are dispersed in the mortar. As the first level of micro-dispersion system in the complex composition of pavement materials, the mastic’s microstructure and characteristics have important impacts on pavement performance. Therefore, this paper first evaluated and studied the microstructure and characteristics of three types of mastic before evaluating the various properties of CRAM.

## 2. Materials and Methods

### 2.1. Materials

#### 2.1.1. Emulsified Asphalt

Cationic slow-setting emulsified asphalt was prepared by colloidal mill with matrix asphalt binder (SsangYong Brand, South Korean) and cationic emulsifier in laboratory. The specified cationic emulsifier for cold recycled pavement was obtained from Paini Chemical Co., Ltd., Binzhou, China. The properties of the matrix asphalt and emulsified asphalt are presented in Table 1 and Table 2, respectively.

#### 2.1.2. RAP

The characteristics of RAP have very important impacts on the performance of the mixture. In this study, RAP was collected from the overhaul milling materials of the Qingdao Jiaozhou Bay Highway. The RAP was sieved according to JTG E42-2005 (Test Methods of Aggregate for Highway Engineering) [20] in order to understand its particle size distribution. The results are listed in Table 3. The maximum and minimum values refer to the range of medium-size gradation for CRAM in JTG F41-2008 (Technical Specifications for Highway Asphalt Pavement Recycling) [19].

**Figure 1:**
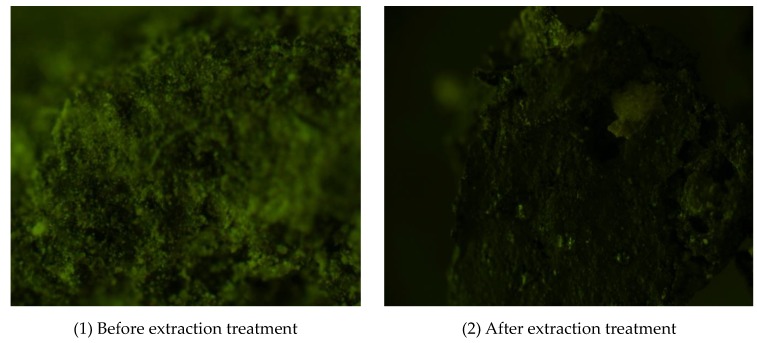
Fluorescence microscopic images of RAP.

As shown in Table 3, the mineral filler and fine aggregate contents were very low. The RAP was then subject to an extraction test and rescreened. The results are presented in Table 3, where PP-AE represents the passing percent after extraction. Compared to the initial RAP gradation, the gradation of RAP after extraction testing was lower, and the mineral filler and fine aggregate contents apparently increased. Furthermore, the microstructure of RAP was investigated by fluorescence microscopy (FM), as shown in Figure 1.

It can be seen that a large amount of mineral filler and fine aggregate was wrapped around the asphalt in the RAP before extraction treatment. After the extraction, the above-mentioned mineral filler and fine aggregates all peeled off from the surface of the RAP. This may explain the lack of fine aggregates in the gradation before the extraction experiment.

The experimental gradation was determined by considering the distribution of particle size in RAP and the specification of the medium-size gradation for CRAM in JTG F41-2008. It should be emphasized that the experimental gradation selected in this article used 100% RAP without any new aggregates being added, as shown in Table 3.

To minimize the influences of gradation differences on the experimental results, the RAP was firstly sieved to 12 grades by a sieve shaker, from 0.075 mm to 26.5 mm. The RAP samples were stored separately by size and later combined into an experimental aggregate of the desired gradation according to the percentage passing each sieve.

#### 2.1.3. Recycled Asphalt from RAP

The recycled asphalt was extracted from RAP, and its properties are listed in Table 4. It can be seen that the penetration of recycled asphalt was 20 and the ductility was only 34.5 cm, which indicates that the asphalt was seriously aged. Despite this, it still has an important impact on the diffusion process of newly added asphalt binder [21].

#### 2.1.4. Cement

For CRAM, cement is commonly used to promote demulsification of emulsified asphalt and to increase the initial strength [22]. Ordinary Portland cement (PO. 32.5; Shanshui Brand, Sunnsy Group, Jinan, , China) was used in this study. Only the part of cement that can pass at the 0.075 mm sieve were used to make cement-residue mastic.

#### 2.1.5. Mineral Filler

In this study, limestone mineral fillers from five different areas (Pingyin, Longqing, Rilan, Changle, and Dongqing) were used, and labeled 1#, 2#, 3#, 4#, and 5#, respectively. Density, hydrophilicity coefficient (H-C), and methylene blue value (MBV) tests were carried out on the five kinds of mineral filler. The results are shown in Table 5.

It can be seen that the density and H-C of the five limestone mineral fillers are not much different, but their MVBs have obvious differences. The MBVs of 2#, 3#, and 4# are significantly larger than those of 1# and 5#, indicating that the first three fillers contain more expansive clay mineral components relatively.

### 2.2. Preparation of Three Types of Mastic

Only the part of mineral fillers that can pass at the 0.075 mm sieve were used to make mineral filler-asphalt mastic and mineral filler-residue mastic.

#### 2.2.1. Mineral Filler-Asphalt Mastic

Five kinds of mineral filler-asphalt mastics were prepared according to filler–binder mass ratios (FBRs) of 0.6, 0.8, 1.0, 1.2, and 1.4, and labeled 1-H, 2-H, 3-H, 4-H, and 5-H, respectively. The FBR refers to the weight ratio of limestone mineral filler to matrix asphalt binder.

#### 2.2.2. Mineral Filler-Residue Mastic

Five kinds of mineral filler-residue mastics were prepared according to FBRs of 0.6, 0.8, 1.0, 1.2, and 1.4, and labeled 1-EH, 2-EH, 3-EH, 4-EH, and 5-EH, respectively. Here, FBR refers to the weight ratio of mineral filler to emulsified asphalt evaporation residue (EAR).

#### 2.2.3. Cement-Residue Mastic

After cement is added to the CRAM, it will hydrate with some of the water in the mixture and form hydration products. In order to simulate the process of cement hydration in the laboratory, a certain proportion of cement was first added to the emulsified asphalt and stirred for 15 min. Then, the mixture stood at room temperature for one day. Finally, the water in the mixture was evaporated by an electric heating sleeve to obtain cement-residue mastic, labeled C-EH.

Referring to the conventional dosages used in actual pavement works, the amounts of emulsified asphalt selected were 3.5, 4.5, 5.5, and 6.5 wt %, respectively, while the cement dosages were 1, 2, 3, and 4 wt %, respectively. The above mass percentages are all relative to the aggregate weights in the mixtures. Combined with the solid content of the emulsified asphalt and the amount of cement, the FBR of the cement-residue mastic was calculated (Table 6). It should be noted that the FBR here is the mass ratio of cement to EAR.

### 2.3. Preparation of CRAM Specimens

The CRAM specimens were fabricated with RAP, cement, emulsified asphalt, and water at certain proportions using a laboratory mixer. CRAM using 100% RAP was designed by modified Marshall Method in China according to JTG F41-2008 and the method including three processes. Firstly, the optimum water content (OWC) of CRAM was determined by the density of samples prepared by heavy hammer while the emulsified asphalt content was determined as 4.0%. Then, the optimum asphalt emulsion content (OEC) was determined by Marshall stability of samples prepared by Marshall test under the OWC. Lastly, the performance of CRAM was examined by indirect tensile strength and moisture damage resistance. During the mix design process, the OEC and OWC were determined as 4.0% and 5.8% by weight of aggregates, respectively. The CRAM specimens were then placed in the ventilated oven with the temperature of 60 °C for 21 days to fully complete the curing process.

For indirect tensile tests and freeze–thaw splitting tests, the loose mixture was compacted into samples with dimensions of 101.6 mm × 63.5 mm using a Marshall compactor. For rutting tests, the loose mixtures were compacted into specimens with dimensions of 300 mm × 300 mm × 50 mm using a wheel-rolling compactor. For dynamic modulus tests, the loose mixture was compacted using a superpave gyratory compactor with 55 cycles to produce cylindrical specimens with dimensions of 150 mm × 170 mm. After the curing period, specimens for the dynamic modulus tests were cored from the centers of the compacted specimens and sawed at each end to dimensions of 100 mm × 150 mm.

### 2.4. Experimental Method

#### 2.4.1. Dynamic Shear Rheological

Dynamic shear rheology tests can simulate traffic conditions well and can be used to study the high-temperature rheological properties of asphalt and mastics. Prior to carrying out viscoelastic tests, strain sweep tests (0.01–20%) were performed using the dynamic shear rheometer (DSR) to determine the linear viscoelastic (LVE) limit for all mastics at 60 °C and 10 rad/s in this study. The LVE strain range was regarded as the strain within 10% reduced value of the maximum complex modulus with increases in the shear strain [23,24]. Then the test was conducted by the DSR with the 25 mm (1 mm gap) parallel plate set-up in the strain-controlled mode.

#### 2.4.2. Indirect Tensile Tests

Indirect tensile tests are used to determine the mechanical properties of an asphalt mixture at a specified temperature and loading rate. They also can be used to evaluate the low-temperature crack resistance of an asphalt mixture. In this study, the experimental temperature was 15 °C, the loading rate was 50 mm/min, and the specimens were immersed in a constant-temperature water tank that was recycled under reflux for not less than 1.5 h before testing.

#### 2.4.3. Freeze–Thaw Splitting Tests

The splitting strength ratios of the specimens before and after water damage were determined to evaluate the water stability of the asphalt mixtures. The specimens were divided into two groups for the tests. The first set of samples was used to measure the splitting strength R_T1_ without freezing and thawing cycles, and the second set was used to determine the splitting strength R_T2_ of the freeze–thaw cycle. The treatment of freeze–thaw cycles was carried out in accordance with the requirements of AASHTO T283 and JTG E20-2011 (Standard Test Methods of Bitumen and Bituminous Mixture for Highway Engineering) [25]. The tensile strength ratio (TSR) can be calculated by Equation (1).

(1) $$\mathrm{TSR}=\frac{R_{T2}}{R_{T1}}\times 100$$

#### 2.4.4. Rutting Tests

Asphalt mixtures have flow and deformation abilities at high temperature due to their viscoelastic performance [26]. Therefore, the repeated loadings of channel traffic and heavy vehicles can cause permanent and irreversible deformation of the pavement [27]. Dynamical stability at 60 °C was adopted to characterize the high-temperature stability of CRAM in this paper. Studies have shown that curing conditions have an important impact on the performance of asphalt mixtures [28]. The tests were carried out according to the requirements of AASHTO T324 and JTG E20-2011. Before the tests, the specimens were initially cured at room temperature for 12 h and then put into the device with the controlled temperature at 60 °C for 48 h. The testing temperature was 60 °C and the wheel pressure was 0.7 MPa.

#### 2.4.5. Dynamic Modulus Tests

**Figure 2:**
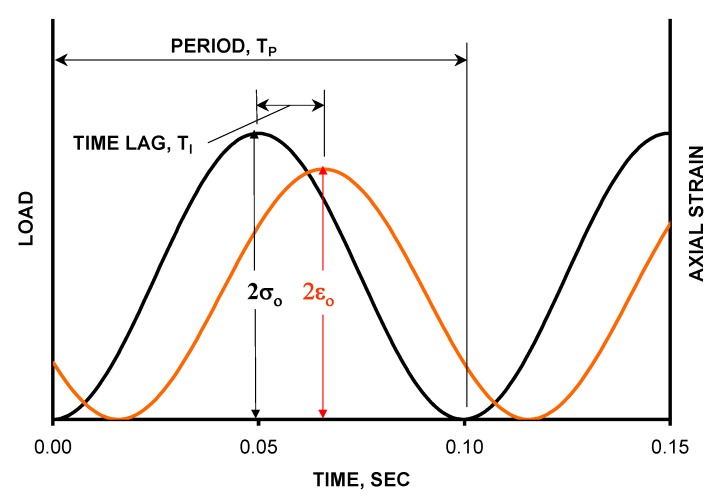
Loading mode of dynamic modulus test.

In the dynamic modulus tests, a sinusoidal axial compressive stress was applied to CRAM specimens at a given temperature and loading frequency. Figure 2 shows the loading mode of the dynamic modulus tests.

According to the AASHTO TP62 testing standard, the testing temperature was set to 20 °C and the dynamic modulus and phase angle were measured at nine different loading frequencies: 25, 20, 10, 5, 2, 1.0, 0.5, 0.1, and 0.01 Hz. Testing at a given temperature should begin at the highest loading frequency and proceed to the lowest one.

The applied stress and resulting recoverable axial strain response of the specimens were measured and used to calculate the dynamic modulus and phase angle according to Equations (2)–(5).

(2) $$\sigma \left(t\right)=\sigma_{0}\sin\omega t$$

(3) ω=2πƒ

(4) $$\emptyset =\frac{T_{i}}{T_{p}}\times 360$$

(5) $$\left|E^{*}\right|=\frac{\sigma_{0}}{\varepsilon_{0}}$$

where, ω is the loading angular frequency (rad/s),

ƒ is the loading frequency (Hz),
*φ* is the phase angle (°),
Ti is the average lag time between stress-strain cycles (s),
T_p_ is the average duration of a stress cycle (s),
σ_0_ is the average peak stress (MPa),
ε_0_ is the average peak strain (mm/mm), and
E* is the dynamic modulus (MPa).

## 3. Results and Discussion

### 3.1. Properties of Mastics

#### 3.1.1. Rheological Properties

**Figure 3:**
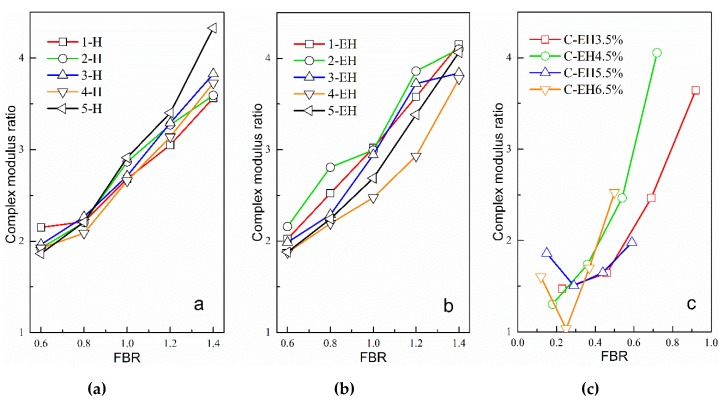
Relationship between mastic modulus and FBR. (**a**) Mineral filler-asphalt mastic (**b**) Mineral filler-residue mastic (**c**) Cement-residue masticAs can be seen in Figure 3, the modulus of the three types of mastics were all higher than those of matrix asphalt or EAR, and the modulus of the mineral filler-asphalt mastic specimen increased linearly with increases in FBR. When the FBR was 0.6, the five kinds of mineral filler doubled the increase, and when the FBR was 1.4, the mineral filler increased the modulus by more than double. The mineral filler-residue mastic exhibited a similar pattern to the mineral filler-asphalt mastic. This result is related to the hardening of inorganic mineral fillers; that is, the addition of mineral filler can increase the elastic recovery modulus of mastics, causing them to exhibit very linear growth relationships, and enhancing the mastics’ resistance to deformation [29].

In order to avoid the influence of differences in the performances of matrix asphalt or EAR on the experimental results, the modulus of three types of mastics (obtained by DSR tests) was divided by the modulus of the matrix asphalt or EAR to obtain a ratio defined as the modulus increase ratio. Figure 3 shows the relationship between the modulus increase ratio and the FBRs of three types of mastics at 60 °C. It should be noted again that the filler–binder mass ratio in Figure 3(1) is the weight ratio of mineral filler to asphalt binder; while in Figure 3(2) it is the weight ratio of mineral filler to EAR; and in Figure 3(3) it is the weight ratio of cement to EAR.

The variation in the cement-residue modulus was more complicated than that of the first two types of mastics. With amounts of emulsified asphalt of 3.5% and 4.5%, the modulus of the mastic increased with increases in FBR. With dosages of 5.5% and 6.5% emulsified asphalt, the modulus of the mastic first decreased and then increased rapidly with increases in FBR, with the lowest value occurring at an FBR of about 0.3. In summary, the modulus-increasing effect of the cement-residue mastic was the most significant. The FBR required to achieve the same modulus increase was smaller than for the first two types of mastics. In other words, when FBR is equal, the modulus of the cement-residue mastic increases more obviously. This is because the cement particles can not only act as a filler, but the formation of hydration products in the mastic greatly promotes the increase of the mastic’s modulus.

**Figure 4:**
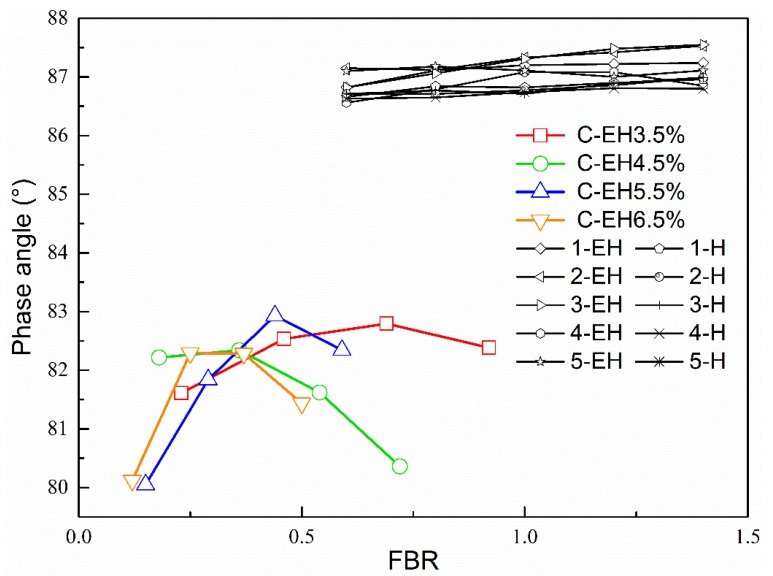
Relationships between mastic phase angles and FBR.

Figure 4 shows that as FBR increased, the changes in the phase angles of the mineral-asphalt mastic and mineral-residue mastic at 60 °C were not significant, and were stable between 86.5° and 87.5°. That is, under high-temperature conditions, the inorganic mineral filler had little effect on the viscoelastic composition ratio of the base asphalt or EAR but improved the modulus remarkably. The phase angle of the cement-residue mastic was lower than that of the first two types of mastics, which showed that the addition of cement could reduce the strain-to-stress response lag time and increase the elastic solid properties of the mastic as a viscoelastic material.

#### 3.1.2. Interface Bonding Performance

The indicator K. Ziegel-B was proposed by Ziegel et al. and can be used to evaluate interfacial interactions. It can be calculated by testing the phase angles of asphalt binders and asphalt mastics [30]. The concept is to consider that the interaction between asphalt binder and mineral fillers can make the fillers absorb some asphalt binder fractions, so the effective filler fraction should be based on the real filler fraction. The correction can be achieved by the B value, which is a generalized interaction parameter that can represent complicated physical and chemical interactions. The larger the B value, the stronger the interfacial interaction.

By experimental observation of many composites, the damp of filling system can be calculated as follows,

(6) $$\tan\delta_{c}=\frac{tan\delta_{m}}{1+\varphi B}$$

So, the B value can be calculated according to Equation (7).

(7) $$B=\frac{\frac{\tan\delta_{m}}{tan\delta_{c}}-1}{\varphi }$$

where, $\delta_{c}$ = phase angle of composite (rad),

φ = volume fraction of filler (volume ratio of filler in asphalt mastics),

$\delta_{m}\;$= phase angle of asphalt binder (rad), and

B = the K. Ziegel-B filler-matrix interaction parameter.

**Figure 5:**
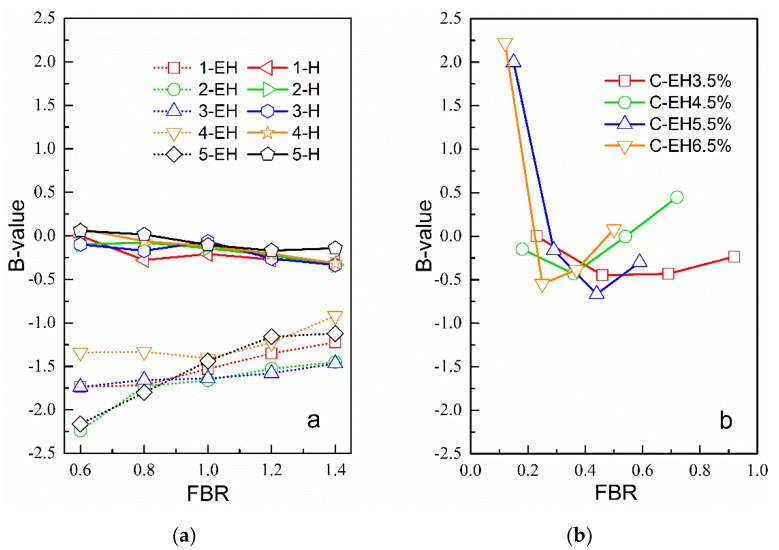
B-values of mastics with (**a**) mineral filler and (**b**) cement.

In the volume fraction conversion, the density of EAR and asphalt is 1 g/cm^3^, the density of mineral filler (<0.075 mm) is 2.65 g/cm^3^, and the density of cement is 3.14 g/cm^3^ [31,32,33]. The interfacial adhesion coefficients (B) of the three types of mastic were calculated according to Equation (7) and the results are shown in Figure 5.

It can be seen that the B-value of the mineral filler-asphalt mastic was higher than that of the mineral filler-residue mastic. The interfacial adhesion coefficient (B-value) of the mineral filler-asphalt mastic decreased with increases in FBR, while for mineral filler-residue mastic it showed a linear increase with FBR.

The interfacial adhesion coefficient of the cement-residue mastic was significantly different from that of the above two kinds of mastics. The B-value of the cement-residue mastic firstly decreased and then increased with increases in FBR. The variations in the samples with the four emulsified asphalt contents were the same. Even when the interface adhesion coefficient was the lowest, it was at the same level as the mineral filler-asphalt mastic. In addition, in most cases, the B-value was higher than that of the above two kinds of mastic. Compared to cement-free mineral filler-residue mastic, the B-value of cement-containing mastic can be several times higher. This shows that the addition of cement can greatly improve the interfacial bonding between binders and fillers in the mastic, thereby improving the water damage resistance and high-temperature stability of CRAM.

#### 3.1.3. Microstructure of Mastics

**Figure 6:**
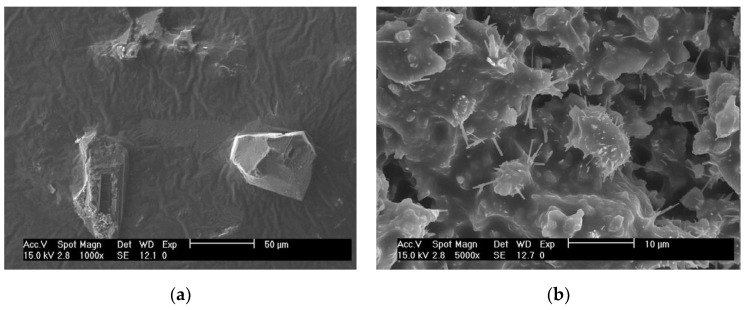
SEM images of (**a**) Mineral filler-asphalt mastic and (**b**) Cement-residue mastic.

It can be seen from the above experimental results that the properties of cement-residue mastic are very different from those of the other two types of mastic consisting solely of mineral filler. So, the microstructure of mineral filler-asphalt mastic and cement-residue mastic were characterized by FESEM (HitachiS4800, Japan), as shown in Figure 6.

It can be seen that the mineral filler-asphalt mastic exhibited a pure particle filling system, while the cement-residue mastic did not and there were a lot of distinct whisker-like hydration products. This is because the cement can react with the water in the mixture after it is added to the CRAM and the hydration products are very complex. The main components of ordinary Portland cement are tricalcium silicate, dicalcium silicate, tricalcium aluminate, tetracalcium aluminate, and calcium sulfate. The main chemical reactions are

2(2CaO·SiO_2_) + 5H_2_O = 3CaO·2SiO_2_·3H_2_O + Ca(OH)_2_

2(2CaO·SiO_2_) + 4H_2_O = 3CaO·2SiO_2_·3H_2_O + Ca(OH)_2_

3CaO·Al_2_O_3_ + 6H_2_O = 3CaO·Al_2_O_3_·6 H_2_O

4CaO·Al_2_O_3_·Fe_2_O_3_ + 2Ca(OH)_2_ + 10H_2_O = 3CaO·Al_2_O_3_·6H_2_O + 3CaO·Al_2_O_3_·Fe_2_O_3_·6H_2_O

3CaSO_4_ + 3CaO·Al_2_O_3_ + 32H_2_O = 3CaO·Al_2_O_3_·3CaSO_4_·32H_2_O

It can be seen from the above chemical reactions that the cement hydration products are mainly alkaline calcium silicate, calcium aluminate, and calcium hydroxide, of which the first two are the main cementing components [34].

When the water-cement ratio changes, the degree of cement hydration will also be different, and a mastic system with a different material composition and microstructure will be formed. Due to the different hydration effects caused by different water-cement ratios, the cement-residue mastic actually consists of complex phases such as unhydrated cement, hydration products, and EAR. The hydration product is in a crystalline state and interwoven with the EAR, and the unhydrated cement particles act as a filler and micro-aggregate in the overall mastic system [35,36]. Therefore, compared with the mineral filler-asphalt mastic and the mineral filler-residue mastic, the modulus and phase angle of the cement-residue mastic changed differently, and the interfacial bonding performance also showed unique characteristics.

As CRAM cannot use a large asphalt content and has a weaker modulus effect caused by fine aggregates and mineral filler, the final strength of CRAM is mainly provided by cement mastic. From this perspective, emulsified asphalt plays an important role in the initial stability of the mixture, while cement hydration has a positive influence on its ultimate strength [37,38].

### 3.2. Effect of Emulsified Asphalt

In combination with the above experimental evaluation of mastic properties and the determined gradation, and using 100% RAP without adding any new aggregate, the influences of emulsified asphalt content on the void ratio, ITS, tensile strength ratio, dynamic stability, and dynamic modulus of CRAM were investigated. The cement dosage was selected as 2.0 wt % and the overall water content was determined to be 5.8% throughout this section, and the free water usage in different mixtures (with different emulsion content) needed to be adjusted based on the change in the amount of emulsified asphalt.

#### 3.2.1. Void Ratio

The void ratio is the main index used to evaluate the compaction of asphalt mixtures. In the process of examining the impact of emulsified asphalt content on the properties of CRAM, the total liquid content should be constant. In this study, the emulsified asphalt contents selected were 2.5, 3.0, 3.5, 4.0, 4.5, 5.0, and 5.5 wt %.

**Figure 7:**
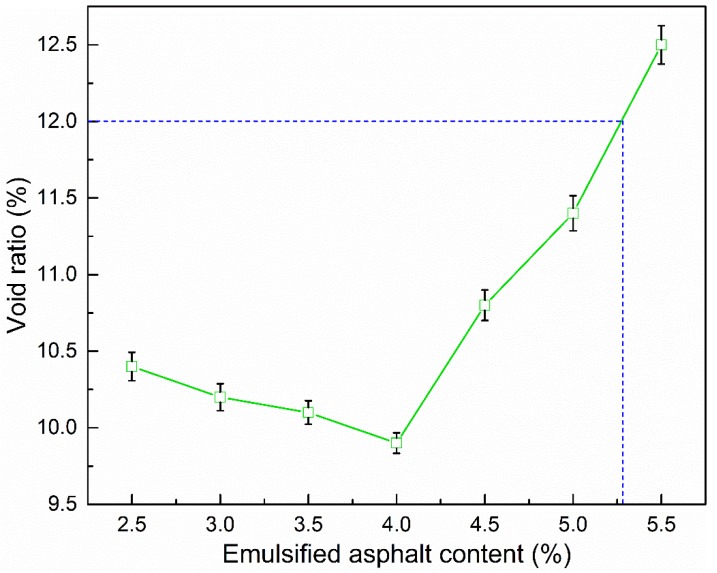
Influence of emulsified asphalt content on void ratio.

As can be seen from Figure 7, when the emulsified asphalt content increased, the void ratio of CRAM initially decreased to a minimum and then began to increase. The main reason is that the change in viscosity for the total liquid has little effect on the inner friction of the asphalt mixture when the added emulsified asphalt content is lower than 4.0% and the total liquid content is constant. At that time, the residue content of total liquid increases, which will decrease the void ratio. Meanwhile, when the emulsified asphalt content continues to increase above 4.0%, the viscosity of total liquid will increase, and then the inner friction of the asphalt mixture becomes higher, which makes compaction of the mixture difficult. It can be seen that as the amount of emulsified asphalt is further increased, the porosity begins to increase correspondingly.

#### 3.2.2. Indirect Tensile Strength

**Figure 8:**
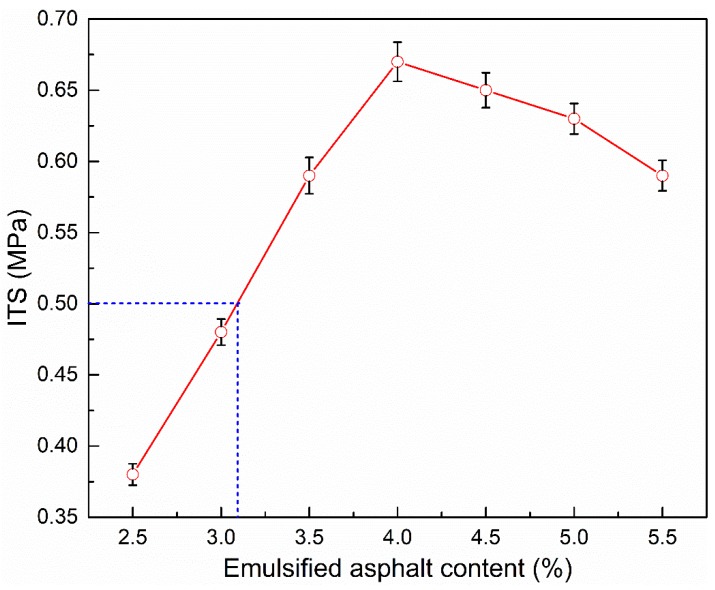
Influence of emulsified asphalt content on ITS.

In this section, the amounts of emulsified asphalt selected were 2.5, 3.0, 3.5, 4.0, 4.5, 5.0, and 5.5 wt %. The influence of emulsified asphalt content on the indirect tensile strength of CRAM are presented in Figure 8.

According to Figure 8, as the amount of emulsified asphalt increases, the ITS of CRAM initially increases to a maximum and then begins to decrease. When the emulsified asphalt content is 4.0%, the ITS is the greatest and the void ratio is the smallest at the same time. This shows that an increase in porosity will hinder the development of ITS.

#### 3.2.3. Tensile Strength Ratio

**Figure 9:**
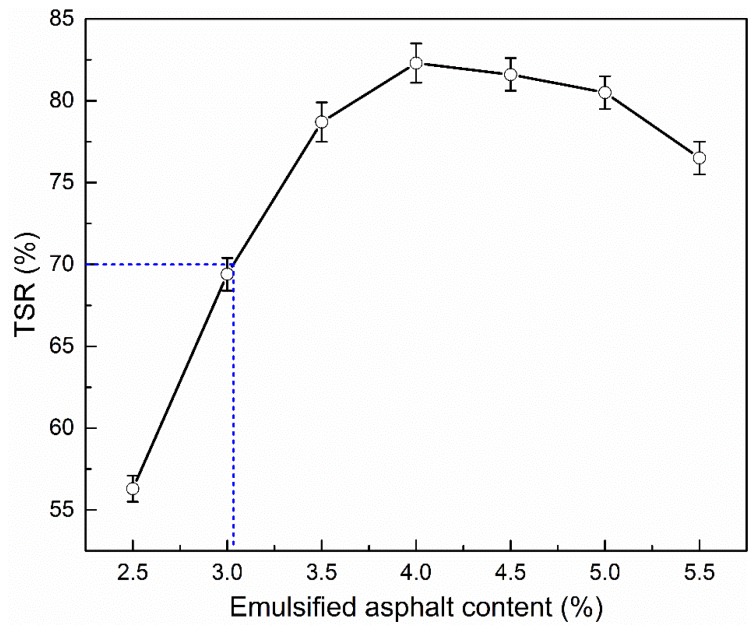
Influence of emulsified asphalt content on TSR.

Figure 9 illustrates the influence of emulsified asphalt content on the TSR of CRAM. It can be seen that the TSR increases at first with increases in the amount of emulsified asphalt and then decreases slightly. This suggests that increasing the emulsified asphalt content could improve the moisture resistance stability to a great extent. On the other hand, it can also be seen that the void ratio has a direct impact on TSR and the TSR increases with the decrease of the porosity. The void ratio of CRAM reaches the minimum value when the emulsified asphalt content is 4.0 wt %, while the TSR reaches the maximum value at the same time. This is because the amount of water that can enter the pores of specimen becomes smaller along with the reduction of void ratio, so the inside of the specimen is less susceptible to water damage during the freeze–thaw treatment. Therefore, the water damage resistance of CRAM is enhanced, which is manifested by an increase in TSR. The technical requirements for CRAM design according to JTG F41-2008 are shown in Table 7.

In addition, it can be seen from Figure 7, Figure 8 and Figure 9 that when using 100% RAP, 2% cement, and 3.1–5.3% emulsified asphalt, the porosity, ITS, and TSR are all fully compliant with the specifications of the relevant standard.

#### 3.2.4. Dynamic Stability

**Figure 10:**
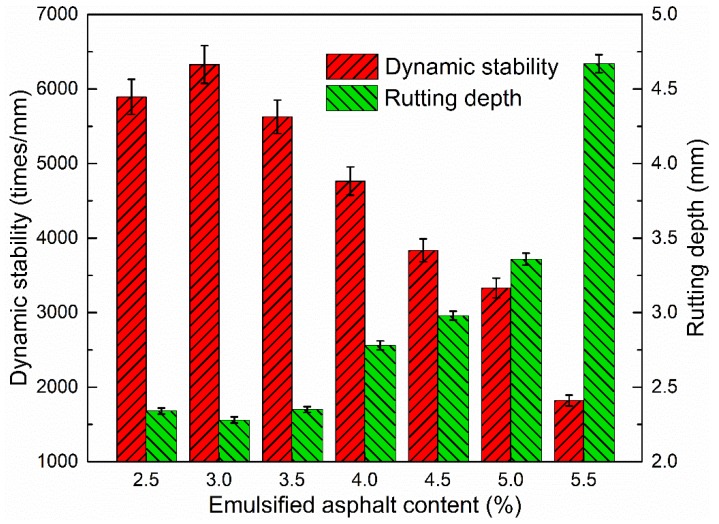
Influence of emulsified asphalt content on dynamic stability and rutting depth.

The dynamic stability can be used to evaluate the high-temperature properties of asphalt mixtures. Thus, the dynamic stability at 60 °C and rutting depth of CRAM were evaluated in terms of emulsified asphalt content, as presented in Figure 10.

It can be concluded from Figure 10 that as the emulsified asphalt content increases, the dynamic stability of CRAM at 60 °C decreases, which is similar to the variation observed for hot mix asphalt [39]. The above results are caused by the asphalt content in CRAM increasing with increases in emulsified asphalt content, which leads to decreases in CRAM inner friction and shear deformation resistance, so the rutting depth tends to increase.

#### 3.2.5. Dynamic Mechanical Analysis

**Figure 11:**
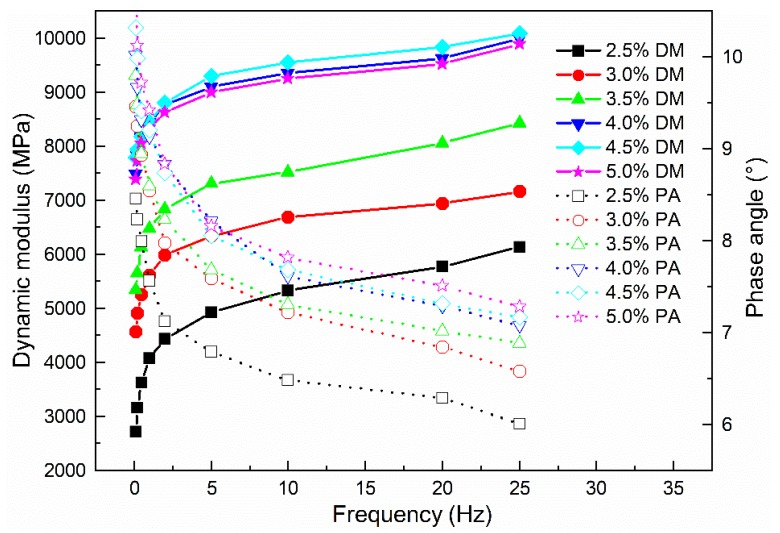
Influence of emulsified asphalt content on mechanical properties of CRAM.

Based on the above investigation of the rheological properties of mastics, the influence of emulsified asphalt content on the rheological properties of CRAM was evaluated. The results are illustrated in Figure 11, where DM and PA represent the dynamic modulus and the phase angle, respectively.

Figure 11 shows that when the amount of emulsified asphalt did not exceed 4.0%, the dynamic modulus of CRAM tended to increase rapidly with increases in emulsified asphalt content. When the amount of emulsified asphalt was more than 4.0%, the dynamic modulus of CRAM tended to be stable and changed little in response to the emulsified asphalt content, while the phase angle still slowly increased.

### 3.3. Effect of Cement

In combination with the above experimental evaluation of the mastics’ properties and determined gradation, 100% RAP without adding any new aggregate was used to investigate the influences of cement content on the void ratio, ITS, tensile strength ratio, dynamic stability, and dynamic modulus of CRAM. The asphalt emulsion content was fixed at 4.0 wt % throughout this section and the optimum water content in different mixtures (with different cement content) needed to be adjusted based on the change in the amount of cement. The OWC was determined by the density of samples prepared by heavy hammer according to T0131 of JTG E40-2007 (Test Methods of Soils for Highway Engineering). In this part, the amounts of cement selected were 0, 1.0, 2.0, 3.0, and 4.0 wt %.

#### 3.3.1. Void Ratio

**Figure 12:**
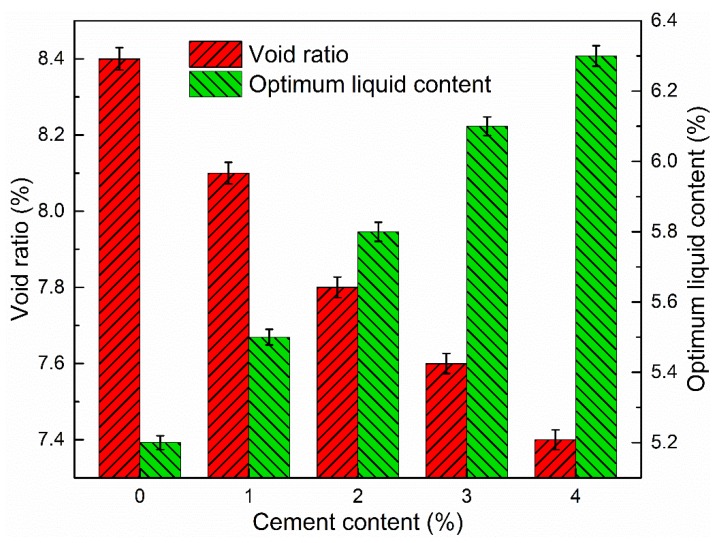
Influence of cement content on void ratio and optimum liquid content.

In order to obtain the optimum compaction of CRAM, the optimum water content should be adjusted according to the cement content. The influence of cement content on the void ratio of CRAM was evaluated, and the results are presented in Figure 12.

It can be seen that the optimum liquid content required for CRAM increased as the amount of cement increased. The main reason is that the cement increased the consistency of the slurry in the mixture and, at the same time, the amount of water used for cement hydration also increased, so more water needed to be added to achieve the best compaction effect. The porosity of CRAM gradually decreased as the cement content increased, but the range of variation was small. The interstitial effect of cement and the impairment of the void ratio by hydrated products lead to this result. Therefore, the amount of cement in CRAM should be controlled within a certain range, generally not more than 2.0 wt %.

#### 3.3.2. Indirect Tensile Strength

**Figure 13:**
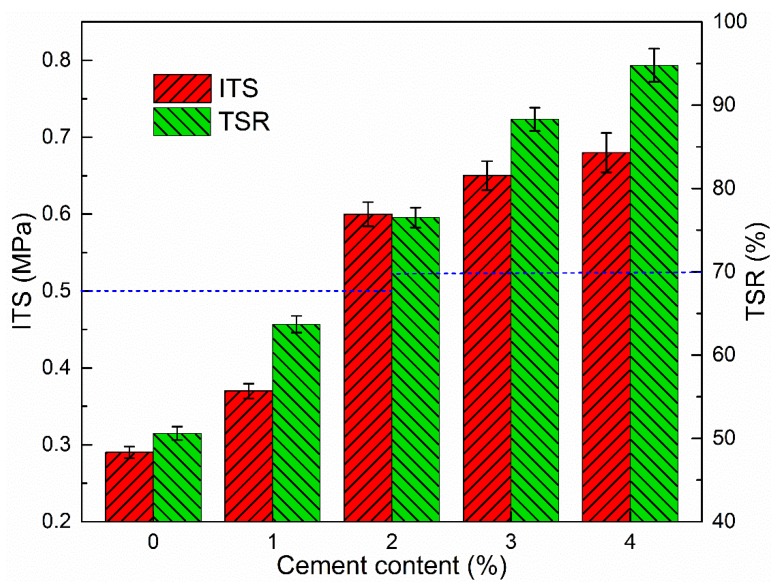
Influence of cement content on ITS and TSR.

As presented in Figure 13, it can be concluded that the ITS of CRAM apparently increased with cement content. This is basically consistent with the variation observed in the cement-residue mastic bonding coefficient, indicating again that the addition of cement can improve the interfacial bonding of aggregate and binders.

#### 3.3.3. Tensile Strength Ratio

The results presented in Figure 13 show that the TSR increased with cement content. The reason is that the porosity of CRAM decreases as the amount of cement increases due to the combined action of cement’s interstitial effect and the impairment of hydrated products. The porosity reduction thereby enhances the ability of the CRAM to resist water damage and is manifested as an increase in TSR, which is used to characterize the ability of the mixture to resist water damage.

In addition, it can be seen from Figure 12 and Figure 13 that with 100% RAP, 4.0% emulsified asphalt, and 2–4% cement, the porosity, ITS, and TSR are all fully compliant with the specifications of the relevant standard.

#### 3.3.4. Dynamic Stability

**Figure 14:**
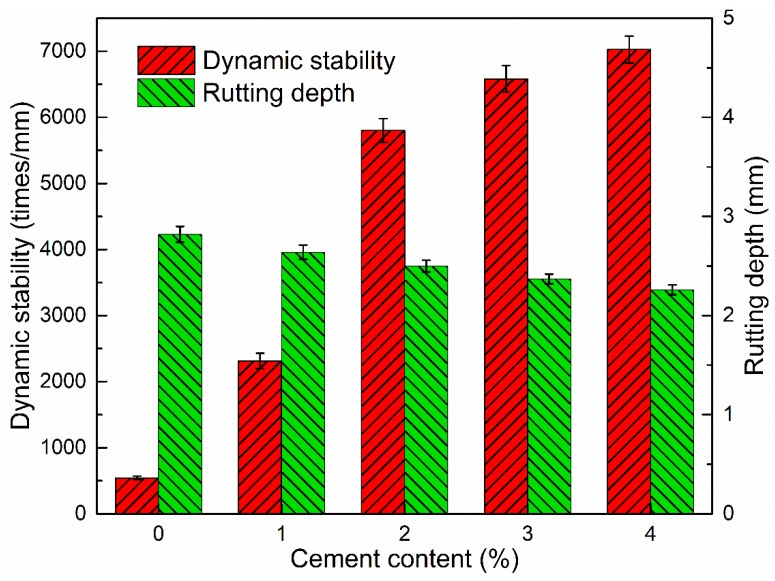
Influence of cement content on dynamic stability and rutting depth.

The results in Figure 14 show that the addition of cement had a great influence on the dynamic stability of CRAM, which varied according to the cement content used. When the cement content was <2.0 wt %, the dynamic stability increased by a large margin, while with >2.0 wt % cement, the increase in dynamic stability with cement content was not as obvious. This relationship is basically consistent with the effect of cement dosage on ITS. It indicates that the addition of cement not only improves the interfacial adhesion of aggregates and binders but also greatly improves the high-temperature stability of CRAM.

#### 3.3.5. Dynamic Mechanical Analysis

**Figure 15:**
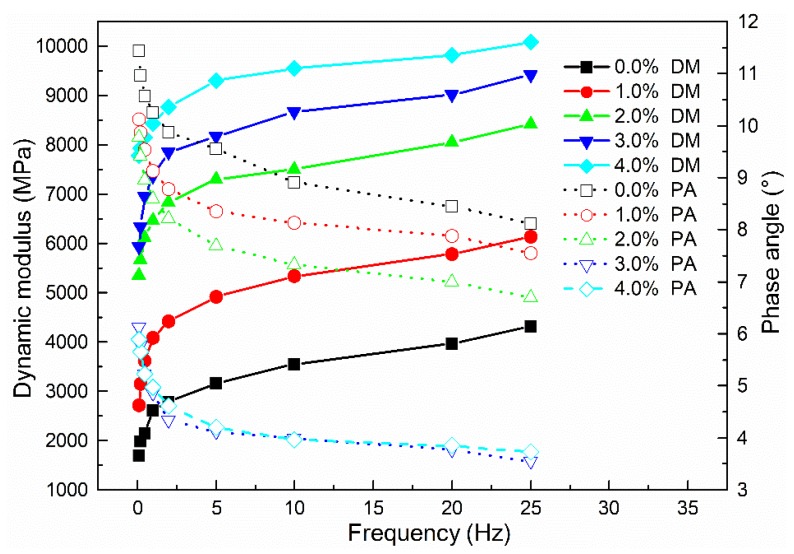
Influence of cement content on mechanical properties of CRAM.

Based on the above investigation of the rheological properties of cement-residue mastic, the influence of cement content on the mechanical properties of CRAM was evaluated. The results are illustrated in Figure 15, where DM and PA represent the dynamic modulus and the phase angle, respectively.

It can be seen that the relationships between cement content and the dynamic modulus and phase angle are different to that for emulsified asphalt. The dynamic modulus increased continuously with increases in cement dosage, with dosages of 1.0–2.0% having the greatest effect. Conversely, the phase angle decreased as the amount of cement increased. When the cement content exceeded 3.0%, the changes in phase angle became very small while the dynamic modulus still increased. Therefore, the addition of cement could reduce the strain-to-stress response lag time and increase the elastic modulus of the CRAM, indicating that the hydration products are beneficial for enhancing the high temperature stability of the CRAM. It is consistent with the previous analysis of the cement-containing mastic’s rheological properties.

## 4. Conclusions

Based on the results described above, the following conclusions can be drawn:

1. Both the modulus of the mineral-filler mastic and mineral-residue mastic increased linearly with the FBR, which is due to the hardening of inorganic mineral filler fillers. The cement-residue mastic’s modulus-increasing effect was most pronounced and its phase angle was lower compared to the first two types of mastics. This is due to the hydration of cement helping to increase the elastic solid properties of the cement-residue mastic.
2. Compared to cement-free mastics, the B-values of cement-containing mastics can be several times higher. This shows that the addition of cement can greatly improve the interfacial bonding between binders and fillers in the mastic, thereby improving the water damage resistance and high-temperature stability of CRAM. It is confirmed by the impact of cement content on the ITS, TSR, and dynamic stability of CRAM.
3. The relationships between cement content and the CRAM’s phase angle are different to that for emulsified asphalt obviously. The addition of cement reduces the phase angle of the CRAM, and it is consistent with the previous results of cement-residue mastic’ rheological properties. This shows the hydration products reduce the strain-to-stress response lag time and increase the elastic modulus of CRAM, and it is beneficial for enhancing the high temperature stability of CRAM.
4. Using 100% RAP, 2.0 wt % cement, and 3.1–5.3 wt % emulsified asphalt, or when using 4.0 wt % emulsified asphalt and 2.0–4.0 wt % cement, the porosity, ITS and TSR of CRAM are all fully compliant with the specifications of JTG F41-2008. Therefore, schemes using CRAM for a subsurface layer of pavement with 100% RAP may be feasible under certain conditions.

## Figures and Tables

**Table 1:** Properties of matrix asphalt.

Parameter		Requirements	Results
Penetration (25 °C; 0.1 mm)		60~80	66
Softening point (°C)		≮46	47.0
Dynamic viscosity (60 °C; Pa·s)		≮180	258
Ductility (10 °C; cm)		≮20	76
Ductility (15 °C; cm)		≮100	>100
Wax content (%)		≯2.2	1.7
Flashing point (°C)		≮260	320
Solubility (%)		≮99.5	99.9
Density (15 °C; g/cm^3^)		Report	1.035
Thin film oven test	Mass change (%)	≯±0.8	–0.022
	Residual penetration ratio (%)	≮61	71
	Residual ductility (10 °C; cm)	≮6	19

**Table 2:** Properties of emulsified asphalt.

Parameter		Requirements [19]	Results
Residue content by evaporation (%)		≮62	63
Sieve residue (1.18 mm; %)		≯0.1	0
Storage stability (5 d, 25 °C; %)		≯5	4
Storage stability (1 d, 25 °C; %)		≯1	0.9
Residual asphalt	Penetration (25°C; 0.1 mm)	50–300	75
	Ductility (15 °C; cm)	≮40	>100

**Table 3:** Results of sieving for RAP

Sieve Size (mm)	0.075	0.15	0.3	0.6	1.18	2.36	4.75	9.5	13.2	16	19	26.5
Passing percent	0.87	1.29	1.86	3.09	4.35	6.95	14.9	44.1	64.3	79.4	91.7	100
PP-AE	6.11	7.60	10.1	13.6	21.2	31.9	61.7	74.6	79.1	89.1	98.2	100
Maximum	8	—	21	—	—	50	65	80	—	—	100	100
Minimum	2	—	3	—	—	20	35	60	—	—	90	100
Experimental gradation	3	4	6	9	15	27	50	74	85	92	97	100

**Table 4:** Properties of recycled asphalt

Parameter	Results
Penetration (25 °C; 0.1 mm)	20
Softening point (°C)	65
Ductility (25 °C; cm)	34.5
Dynamic viscosity (60 °C; Pa·s)	544

**Table 5:** Properties of the mineral fillers

Parameter	1#	2#	3#	4#	5#
Density (g.cm^−3^)	2.673	2.657	2.618	2.711	2.683
H-C	0.671	0.685	0.652	0.752	0.69
MBV (g/kg)	1	2.25	2.75	2.5	0.75

**Table 6:** Production parameters of cement-residue mastics

Mastic Type	Asphalt Emulsion (%)	Cement (%)	FBR
E-CH3.5%	3.5	0.5	0.23
	3.5	1	0.46
	3.5	1.5	0.69
	3.5	2	0.92
E-CH4.5%	4.5	0.5	0.18
	4.5	1	0.36
	4.5	1.5	0.54
	4.5	2	0.72
E-CH5.5%	5.5	0.5	0.15
	5.5	1	0.29
	5.5	1.5	0.44
	5.5	2	0.59
E-CH6.5%	6.5	0.5	0.12
	6.5	1	0.25
	6.5	1.5	0.37
	6.5	2	0.5

**Table 7:** Technical requirements for CRAM design

Parameter	Requirements	
	Pavement Base or Subbase	Pavement Subsurface Layer
Void ratio (%)	9–12	9–12
15 °C ITS (MPa)	≮0.40	≮0.50
TSR (%)	≮70	≮70
